# Three-dimensional Monte Carlo-based voxel-wise tumor dosimetry in patients with neuroendocrine tumors who underwent ^177^Lu-DOTATOC therapy

**DOI:** 10.1007/s12149-020-01440-3

**Published:** 2020-02-29

**Authors:** Th. I. Goetz, E. W. Lang, O. Prante, A. Maier, M. Cordes, T. Kuwert, P. Ritt, Christian Schmidkonz

**Affiliations:** 1 Department of Nuclear Medicine, Friedrich-Alexander University Erlangen-Nürnberg (FAU), Erlangen, Germany; 2 Pattern Recognition Lab, Friedrich-Alexander University Erlangen-Nürnberg (FAU), Erlangen, Germany; 3 Biophysics, University of Regensburg, Regensburg, Germany; 4 Clinic of Nuclear Medicine, University of Erlangen-Nuremberg, Ulmenweg 18, 91054 Erlangen, Germany

**Keywords:** Monte Carlo simulations, Dosimetry, ^177^Lu-DOTATOC, Neuroendocrine tumors

## Abstract

**Background:**

Patients with advanced neuroendocrine tumors (NETs) of the midgut are suitable candidates for ^177^Lu-DOTATOC therapy. Integrated SPECT/CT systems have the potential to help improve the accuracy of patient-specific tumor dosimetry. Dose estimations to target organs are generally performed using the Medical Internal Radiation Dose scheme. We present a novel Monte Carlo-based voxel-wise dosimetry approach to determine organ- and tumor-specific total tumor doses (TTD).

**Methods:**

A cohort of 14 patients with histologically confirmed metastasized NETs of the midgut (11 men, 3 women, 62.3 ± 11.0 years of age) underwent a total of 39 cycles of ^177^Lu-DOTATOC therapy (mean 2.8 cycles, SD ± 1 cycle). After the first cycle of therapy, regions of interest were defined manually on the SPECT/CT images for the kidneys, the spleen, and all 198 tracer-positive tumor lesions in the field of view. Four SPECT images, taken at 4 h, 24 h, 48 h and 72 h after injection of the radiopharmaceutical, were used to determine their effective half-lives in the structures of interest. The absorbed doses were calculated by a three-dimensional dosimetry method based on Monte Carlo simulations. TTD was calculated as the sum of all products of single tumor doses with single tumor volumes divided by the sum of all tumor volumes.

**Results:**

The average dose values per cycle were 3.41 ± 1.28 Gy (1.91–6.22 Gy) for the kidneys, 4.40 ± 2.90 Gy (1.14–11.22 Gy) for the spleen, and 9.70 ± 8.96 Gy (1.47–39.49 Gy) for all ^177^Lu-DOTATOC-positive tumor lesions. Low- and intermediate-grade tumors (G 1–2) absorbed a higher TTD compared to high-grade tumors (G 3) (signed-rank test, *p* =  < 0.05). The pre-therapeutic chromogranin A (CgA) value and the TTD correlated significantly (Pearson correlation:  = 0.67, *p* = 0.01). Higher TTD resulted in a significant decrease of CgA after therapy.

**Conclusion:**

These results suggest that Monte Carlo-based voxel-wise dosimetry is a very promising tool for predicting the absorbed TTD based on histological and clinical parameters.

## Introduction

Neuroendocrine tumors (NETs) are defined as epithelial neoplasms with predominant neuroendocrine differentiation that can arise from neuroendocrine cells throughout the body [1]. Data from the Surveillance, Epidemiology, and End Results (SEER) database suggest that NETs are more prevalent than previously reported with 51% of NETs arising from the gastrointestinal tract, 27% from the lungs, and 6% from the pancreas [2, 3]. NETs of the midgut commonly metastasize to the liver, the mesentery, and the peritoneum. Clinically, they are regarded as functional if they are associated with symptoms of hormonal hypersecretion, the so-called carcinoid syndrome, or non-functional if they are not associated with hormonal hypersecretion [4]. First-line systemic therapy is primarily based on somatostatin analogs, which significantly lengthen time to tumor progression and improve control of hormonal secretion [5, 6]. Besides everolimus, a potent inhibitor of mammalian target of rapamycin (mTOR), for the treatment of non-functional NETs, there have as yet been no standard second-line systemic treatment options [3, 7]. However, the recent United States’ Food and Drug Administration (FDA) approval of ^177^Lu-DOTATATE for the treatment of somatostatin receptor (SSTR)-positive gastroenteropancreatic tumors, based on the results from the phase 3 Neuroendocrine Tumors Therapy trial (NETTER-1), opens new perspectives for the treatment of NETs [4]. Furthermore, a phase 3 trial regarding the safety and efficacy of ^177^Lu-DOTATOC peptide radionuclide receptor therapy (PRRT) compared to targeted molecular therapy with Everolimus is underway (ClinicalTrials.gov identifier NCT03049189). ^177^Lu emits beta particles with a maximal energy of 498 keV, a maximal particle range of 2 mm and has a physical half-life of 6.7 days. Besides beta particles, it also emits gamma photons, which can be directly used for uptake quantification by serial scintigraphy and SPECT [8]. Most of the clinical protocols rely on empirical criteria for choosing the administered activity and the number of cycles [9]. Special emphasis has to be placed on the absorbed doses for kidney and bone marrow, since they are considered as the dose-limiting organs in ^177^Lu-PRRT [10, 11]. Due to the large inter- and intra-patient as well as intra-lesion variability of tumor uptake in PRRT of NETS [12], it is of utmost importance to improve individualized therapy planning. Therefore, methods for accurate dosimetry of tumorous- and non-tumorous tissue and determination of predictive factors that are associated with high uptake of radiolabeled somatostatin analogues in NETs are needed. As yet, only a few previously conducted studies reported the use of the Medical Internal Radiation Dose (MIRD) scheme and the unit density sphere model from Olinda for calculation of tumor-absorbed doses in ^177^Lu-DOTATATE therapy [9, 13] and in ^177^Lu-DOTATOC therapy [14]. In the present study, we used a novel, three-dimensional approach to tumor dosimetry based on SPECT/CT and Monte Carlo simulations to determine total tumor dose (TTD). Furthermore, we sought to identify factors that are associated with a high TTD in patients with SSTR-positive NETs undergoing ^177^Lu-DOTATOC therapy and correlated TTD with changes in serum levels of the tumor marker chromogranin A after therapy.

## Materials and methods

### Patient selection and inclusion criteria

From our clinical database fourteen patients (11 men, 3 women, 62.3 ± 11.0 years of age) with histologically confirmed, unresectable or metastatic NETs of the midgut, who underwent a total of 39 cycles of ^177^Lu-DOTATOC therapy (mean 2.8 cycles, SD ± 1 cycle) between September 2015 and July 2017 were retrospectively enrolled in this study. NETs were assessed as low grade (G 1) if the Ki67 index was 0–2%; intermediate grade (G 2) if the Ki67 index was 3–20%; and high grade (G 3) if the Ki67 index was greater than 20% [4, 15]. In our patient cohort, tumor grade was on average 2.3 (G 1–3). Dosimetry was performed at the first cycle of ^177^Lu-DOTATOC therapy. CgA values were measured at two time points, before and after the last cycle of therapy with a mean time difference between both measurements of 3.9 months (patient characteristics are provided in Table 1). Intense SSTR expression of NETs and their metastases had been verified before therapy by ^68^ Ga-DOTATATE PET/CT or tektrotyd (^99m^Tc-EDDA/HYNIC-Tyr^3^-octreotide) scintigraphy. Preceding treatment was allowed including octreotide/lanreotide (> 4 weeks prior to PRRT), radiation therapy and cytotoxic chemotherapy (> 1 month prior to PRRT). Karnofsky performance status > 50% with adequate bone marrow and renal function (white blood cell count > 3 × 10^3^/µL, red blood cell count > 3 × 10^6^/µL, platelets > 80 × 10^3^/µL and creatinine < 2.0 mg/dL) was required. This retrospective study was performed according to the guidelines of the IRB under the auspices of the Bavarian law concerning hospitals (Bayerisches Krankenhausgesetz 27(4)).

**Table 1:** Patient characteristics

Patients	Age (years)	Grading	CgA (µg/L) before first cycle of therapy	CgA (µg/L) after last cycle of therapy	Time difference of chromogranin A determination (months)	Number of ^177^Lu-DOTATOC cycles
1	45	3	494	1026	5	4
2	66	3	1121	706	4	4
3	54	1	661	1956	4	2
4	67	2	1352	1473	6	3
5	78	2	34	31	4	3
6	54	2	54	77	4	3
7	67	2	597	1748	2	2
8	54	2	1319	682	4	4
9	77	2	224	246	1	1
10	52	3	46	83	5	2
11	52	3	76	47	4	3
12	71	2	51	51	3	2
13	49	3	139	214	2	2
14	79	2	289	2413	7	4
Mean	62	2.3	589.8	768.1	3.9	2.8

### Radiosynthesis and administration of ^177^Lu-DOTATOC

The radiosynthesis of ^177^Lu-DOTATOC was performed in-house in the GMP-compliant clean room facilities of the radiopharmacy at the Nuclear Medicine Clinic of the Erlangen University Hospital, following the general procedure as previously described [16]. DOTATOC acetate (1.0 mg) was purchased from ABX (Advanced Biochemical Compounds GmbH, Radeberg, Germany) and reconstituted in 0.5-mL acetate buffer [0.4 M, pH 4–5, containing gentisic acid (7 mg/mL)] under aseptic conditions and aliquots of the DOTATOC stock solution (100 µg in 1-mL acetate buffer) were stored in sterile vials at − 20 °C. Non-carrier-added [^177^Lu]LuCl_3_ (6–8 GBq in 0.04-M HCl, 0.2 mL) was purchased from ITG (Isotope Technologies Garching GmbH, Garching, Germany). Briefly, the radiolabeling is performed by the addition of DOTATOC (100 µg in 1-mL acetate buffer) to the vial of [^177^Lu]LuCl_3_ and heating at 95 °C for 30 min at a final pH of 3.5–4.0. ^177^Lu-DOTATOC was obtained in a radiochemical purity of > 95% and was formulated with sterile saline solution in a total volume of about 10 mL that was further diluted for intravenous infusion.

Patients were infused intravenously with an average of 6532 ± 449 MBq (range 5773–7265 MBq) ^177^Lu-DOTATOC in physiological saline (100 mL) over a period of 30 min. For renal protection, an intravenous amino acid solution was administered concomitantly starting 30 min before infusion of the radiopharmaceutical.

### Imaging procedure

For dose calculation, several 3D datasets were used. All images were acquired on a hybrid Siemens Symbia T2 SPECT/CT. The acquisition and reconstruction were done based on our standard quantitative ^177^Lu protocol, which is in detail described in Ref. [17]. For this, only the key points of the protocol are listed in the following:

*SPECT*

- Calibrated to kBq/mL based on phantom measurements.
- Medium energy collimator.
- 3° angular sampling, 60 stops (2 projections) for 15 s, 15-min total dwell time.
- Iterative ordered subset expectation maximization (OSEM) reconstruction of the 208 keV photopeak data with 16 iteration 8 subsets, matrix 128 × 128.
- Point-spread-function modelling in reconstruction.
- Triple energy window-based scatter correction.
- CT-based attenuation correction (CT taken from 24 h p.i. SPECT/CT).
- No post-reconstruction smoothing.

*CT*

- Slice collimation of 2 × 5 mm, pitch of 1.8, time per rotation of 0.8 s, tube voltage of 130 kVp, Siemens CareDose 4D tube-current modulation with 30 mAs reference.
- Filtered Back Projection reconstruction with B08s and B41s Kernels, 512 × 512 matrix, 2:5 mm slice thickness.
- B08s image was used for attenuation correction of the SPECT data.
- Down-sampling of B41s to match the lower resolution of the SPECT image (4.79 × 4.79 × 4.79 mm^3^).

### Pharmacokinetics and dosimetry

The decline of radioactivity in the source region is determined by nuclear disintegration and metabolic turnover and can be followed by SPECT imaging. Thus, four SPECT images were recorded at time points *t* = 4 h, 24 h, 48 h and 72 h after administering the radiopharmaceutical. From this series of images, related activities as function of time $a(r_{S} ,t)$ can be deduced and approximated by a model time–activity curve (TAC). The latter is integrated over time to estimate the corresponding time-integrated activity (TIA) $A(r_{S} )$, i.e., the number of radioactive decay events which had happened during the time span considered [18]. By multiplying the estimated TIA with a proper time-independent dose kernel $k(r_{T} \leftarrow r_{S} )$, the energy dose absorbed in the region-of-interest can be computed. Empirically, a large difference is observed if the computation of the absorbed dose is performed either for the entire ROI or separately for each voxel comprising the ROI. This discrepancy mainly stems from the error incurred during the estimation of the TIA. To alleviate this problem, in this study, the TIA was estimated as follows: first, the radioactivity at source locations $r_{{\text{s}}} , s \in S$ was summed up over the entire source region $S$ for the four above-mentioned time points, and the time dependence was then modeled by a mono-exponential function $a(r_{S} ,t) = a(r_{S} ,0){\text{e}}^{{\frac{ - t}{{\tau_{S} }}}}$. The two parameters of this model function were adapted with a least squares fit to the measured activities at the four above-mentioned time points. Alternatively, the activity was determined voxel-wise for all measured time points and an exponential function was adapted to the voxel-wise time-dependent activities according to $a(r_{s,v} ,t) = a(r_{s,v} ,0){\text{e}}^{{\frac{ - t}{{\tau_{s} }}}}$ yielding a pair of parameters $a(r_{s,v} ,0),\tau_{s}$ for every voxel comprising the ROI [18]. Corresponding TIAs were computed by integrating the modeled TACs over time. Finally, the resulting integrated activity of every voxel belonging to the ROI was normalized by the total number of disintegrations within that entire ROI.

Besides the TIA, also the dose kernel needs to be determined before the absorbed dose can be estimated. In this study, the dose kernel has been computed in two different ways. Either the normalized map of voxel-wise TIAs or the voxel-wise mass density distribution, obtained from electron density distributions of an X-ray CT, was fed into a Monte Carlo simulation to estimate the related absorbed energy dose distribution in the interesting target region. Alternatively, following the standard MIRD protocol, the patient-specific mass density map was replaced in the Monte Carlo simulations by data from a standard phantom. Consequently, the differences between the methods are small. The ROIs were defined manually on the fused SPECT/CT images of the kidneys, the spleen and tracer-positive tumor lesions by an experienced nuclear medicine physician.

### Averaged tumor dose

**Fig. 1:**
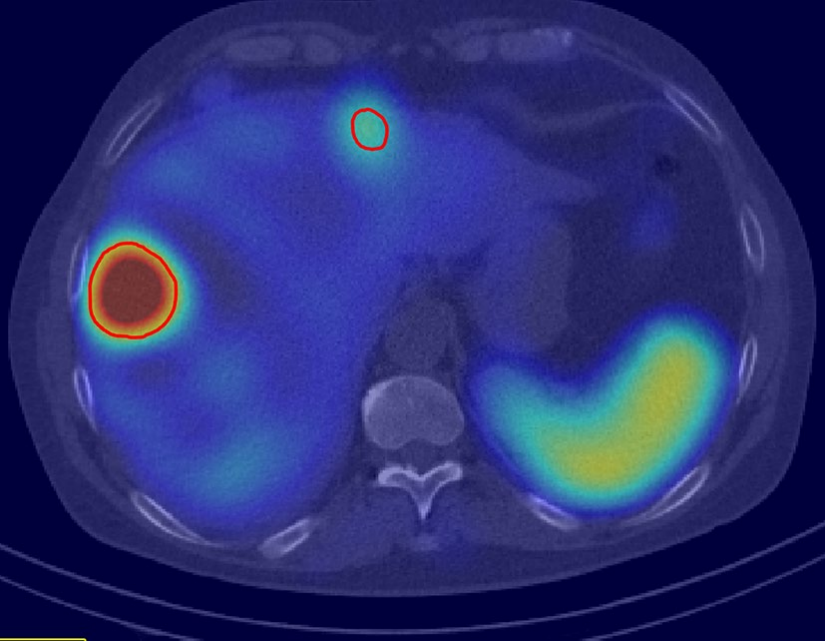
SPECT/CT fusion imaging of a 54-year-old patient with a G2 neuroendocrine tumor suffering from several liver metastases. Regions of interests were drawn surrounding each tracer-positive liver metastasis. Voxel-wise dose values of the right lateral liver metastasis are presented in Fig. 2

**Fig. 2:**
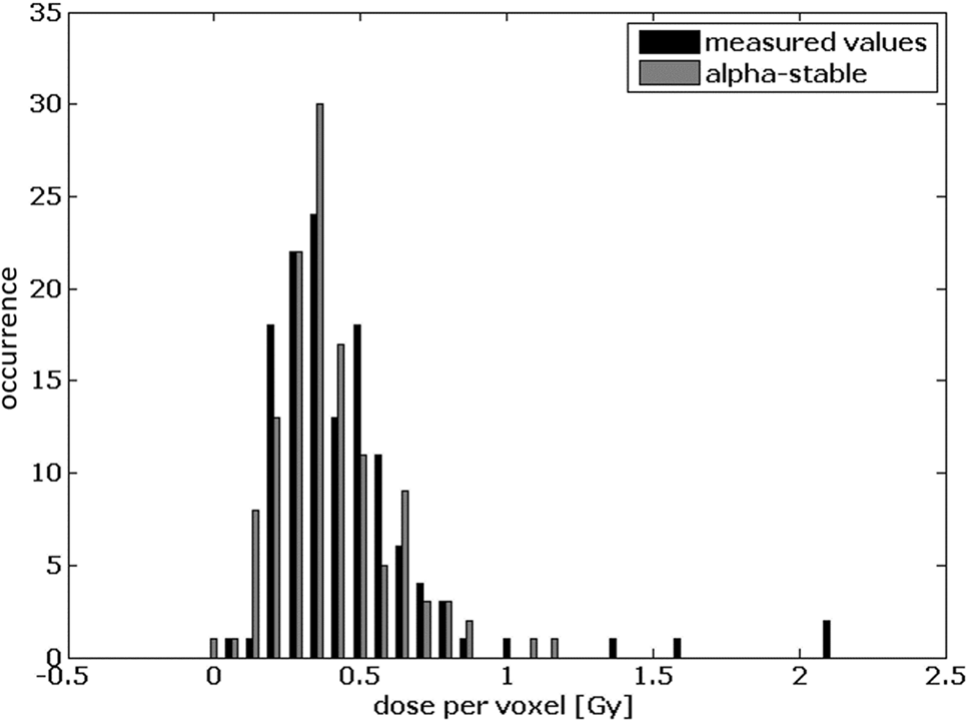
Voxel-wise dose values based on full Monte Carlo simulations for the right lateral liver metastasis from Fig. 1. The alpha-stable distribution is illustrated as gray histogram. Outliers can be seen at 1.6 Gy and 2.1 Gy

To determine the average tumor dose value, a region of interest (ROI) was defined on a fused SPECT/CT by a nuclear medicine physician including all tracer-positive lesions suggestive for tumor (see Fig. 1 for a representative example). The distribution of dose values within a ROI of a liver metastasis is illustrated in Fig. 2. The histogram is not symmetric as it would be for a Gaussian distribution. Rather, the distribution obtained is asymmetric and heavy tailed. This kind of distribution can often be observed in biological systems and can be approximated by an alpha-stable distribution (as also illustrated in Fig. 2).

An asymmetric alpha-stable distribution [19, 20] is characterized by four parameters instead of two parameters of a Gaussian distribution:

$$\varphi (\omega ) = \left\{ {\begin{array}{*{20}c} {{\text{e}}^{{ - \left| {\gamma \omega } \right|^{\alpha } \left[ {1 - i\,{\text{sign}}(\omega )\beta \tan \left( {\frac{\pi \alpha }{2}} \right)} \right] + i\mu \omega }} ,} & {{\text{for}}\,(\alpha \ne 1)} \\ {{\text{e}}^{{ - \left| {\gamma \omega } \right|\left[ {1 + i\,{\text{sign}}(\omega )\frac{2}{\pi }\beta \log \left( {\left| \omega \right|} \right)} \right] + i\mu \omega }} ,} & {{\text{for}}\,(\alpha = 1)} \\ \end{array} } \right..$$

Here $\alpha \in (0,2]$ denotes the impulsiveness, $\beta \in [ - 1, + 1]$ the skewness, $\gamma > 0$ the scale parameter for dispersion and $\mu$ the location parameter, which can be seen as the equivalent to the mean value in a Gaussian distribution.

To get rid of the outliers, the Mahalanobis distance is used, which is unit-less, scale invariant and takes into account the two-point correlations of the data set [21, 22]. This distance measure proves for each measured dose value that it belongs to the assumed statistic or not. Afterwards, the location parameter of the distribution of all dose values with a Mahalanobis distance smaller than one was determined.

### Total tumor dose

Number and location of the patients tumor lesions are given in Table 2. The TTD was calculated according to the following formula:

$${\text{Total}}\,{\text{tumor}}\,{\text{dose}} = \frac{{\mathop \sum \nolimits_{i = 1}^{n} D_{{{\text{tumor}},i}} \cdot V_{{{\text{tumor}},i}} }}{{\mathop \sum \nolimits_{i = 1}^{n} V_{{{\text{tumor}},i}} }},$$

where $D_{{{\text{tumor}}}}$ is the mean tumor value per lesion, $V_{{{\text{tumor}}}}$ the tumor volume and $n$ the number of lesions.

**Table 2:** Organ and tumor averaged dosimetry results per cycle

Patient	Kidney	Spleen	Liver lesion (*n*)	Lymph node (*n*)	Bone lesion (*n*)	Visceral (*n*)	Pancreas (*n*)	Total tumor dose (Gy)
1	2.24	1.89	5.79 (7)					5.79
2	3.13	4.60	8.35 (7)			4.88 (1)		7.50
3	4.71	10.04	44.82 (21)	3.24 (7)		0.88 (1)		39.49
4	2.58	2.69		10.27 (2)	2.49 (4)			8.88
5	3.55	3.04	5.75 (22)					5.75
6	2.90	2.81	3.60 (10)					3.60
7	3.76	1.14	9.56 (9)			2.14 (1)		9.48
8	2.55	3.51	14.55 (32)				6.32 (1)	14.48
9	6.22	6.01				7.10 (1)		7.10
10	3.07	2.88	2.48 (5)					2.48
11	2.53	2.02		1.46 (3)		1.48 (1)		1.47
12	2.71	3.68	12.72 (4)			6.95 (1)		11.17
13	1.91	6.13	6.91 (38)			6.93 (1)	5.23 (1)	6.91
14	5.93	11.22		7.07 (4)	12.55 (14)			11.73
Mean	3.41	4.40	11.45 (155)	5.51 (16)	7.52 (18)	4.34 (7)	5.78 (2)	9.70 (198)

The number of tracer-positive tumor lesions is given in brackets

### Statistics

For the determination of correlations, Pearson’s correlation coefficient *ρ* was calculated. Differences between two groups were evaluated using a Wilcoxon rank-sum test. For all analyses, a *p* value < 0.05 was considered significant. All statistical analyses were performed using Matlab version R2012b (The Math Works Inc., Natick, MA, USA).

## Results

**Fig. 3:**
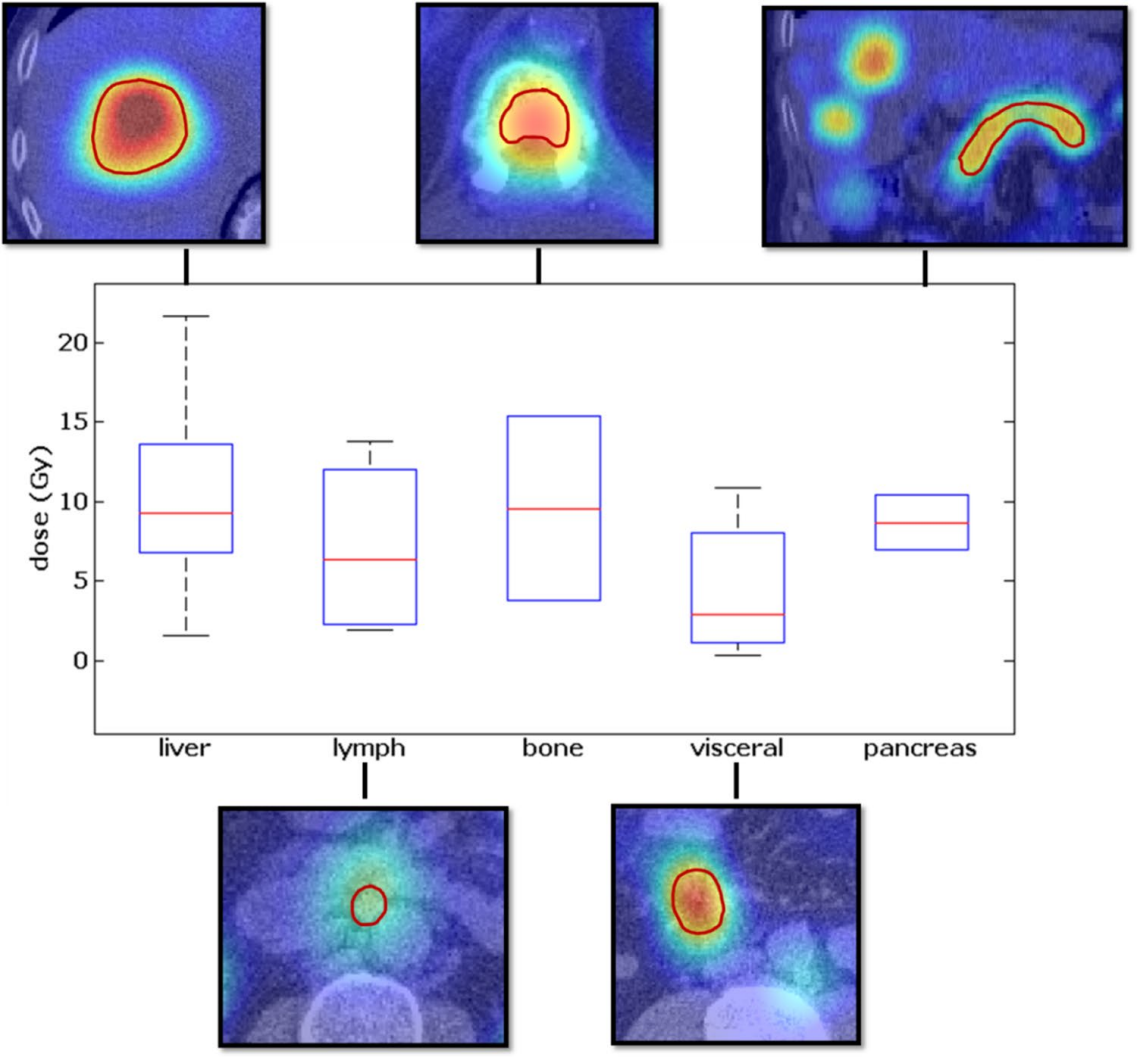
Average dose values for subgroups of tracer-positive tumor lesions and representative fused axial SPECT/CT images

The determined dose values for all organs and tumor lesions are provided in Table 2. Average dose values for subgroups of tracer-positive tumor lesions and representative fused axial SPECT/CT images are illustrated in Fig. 3. Average dose for the kidneys per cycle was 3.41 ± 1.28 Gy (1.91–6.22 Gy), for the spleen 4.40 ± 2.90 Gy (1.14–11.22 Gy), and for all 198 tracer-positive tumor lesions 9.70 ± 8.96 Gy (1.47–39.49 Gy). Average injected activity was 6,532 ± 449 MBq (range 5773–7265 MBq). The dose values per injected activity were 0.52 ± 0.20 mGy/MBq (0.30–0.97 mGy/MBq) for the kidneys, 0.67 ± 0.41 mGy/MBq (0.20–1.62 mGy/MBq) for the spleen, and 1.46 ± 1.26 mGy/MBq (0.20–5.60 mGy/MBq) for the TTD. The mean half-life for the kidney was 67.4 h (27.4–242.6 h) and for the tracer-positive tumor lesions 61.3 h (28.6–416.1 h).

**Fig. 4:**
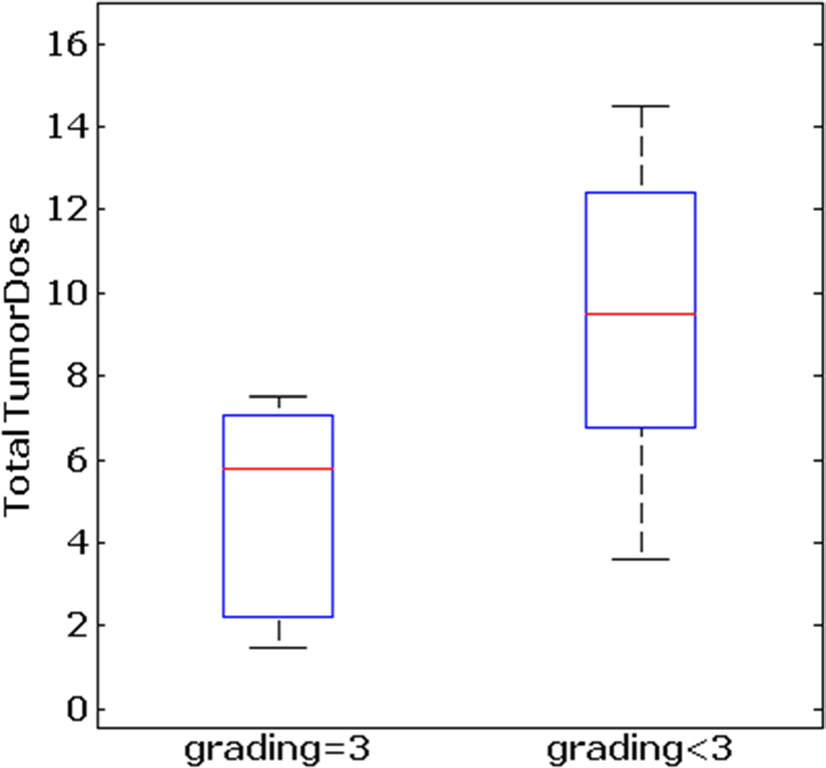
Total tumor dose values are significantly higher in neuroendocrine tumors with a grading smaller than 3 compared to NETs with a grading of 3 (sign-rank test, *p* = 0.042)

**Fig. 5:**
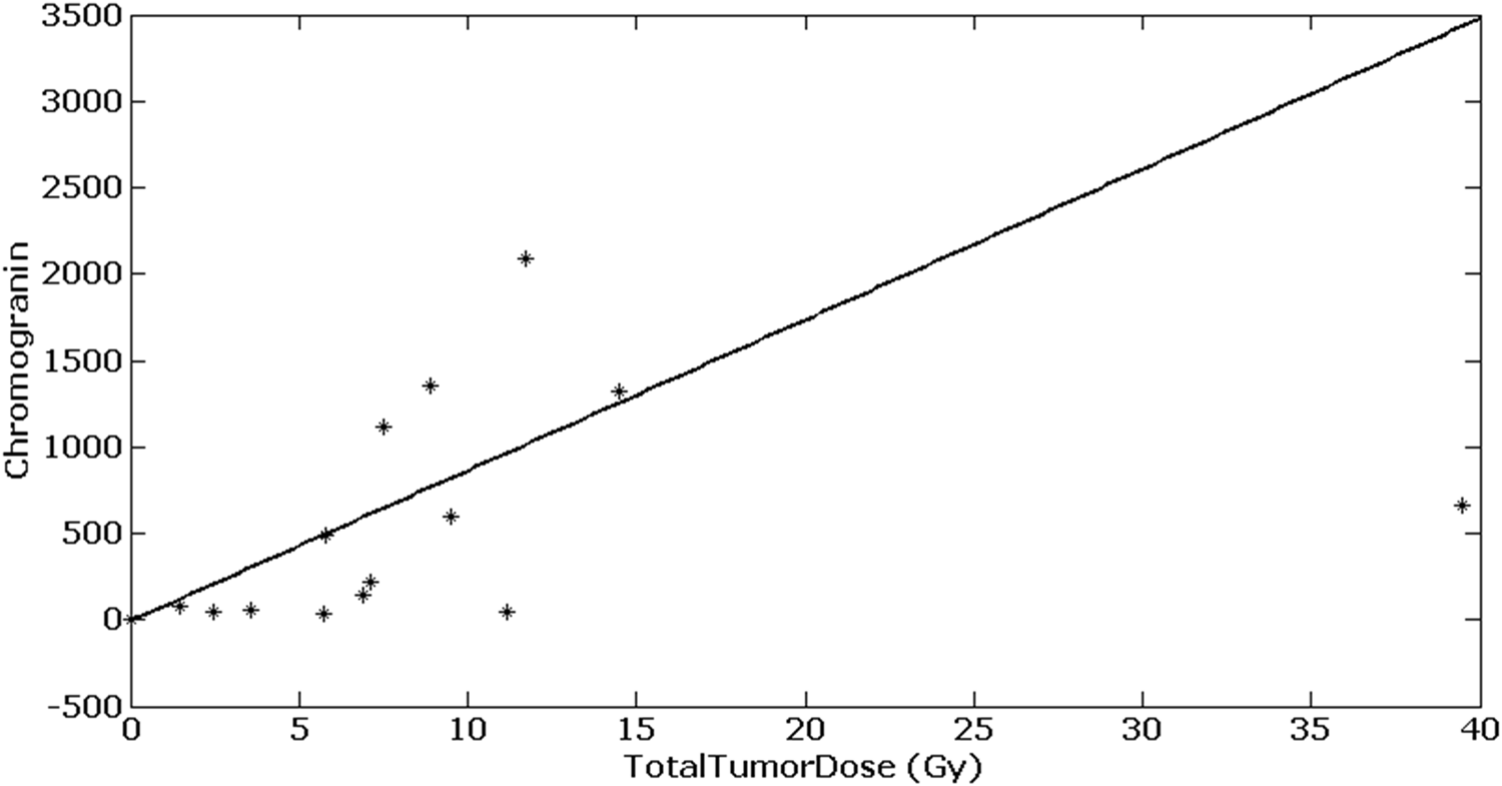
Linear dependency of pretherapeutic chromogranin A values and patient-specific total tumor dose fitted by a linear function (black line) (Pearson correlation: = $\rho$ 0.67, *p* = 0.01)

Low- and intermediate-grade tumors (G 1–2) absorbed a higher TTD compared to high-grade tumors (G 3) (signed-rank test, *p* < 0.05) (see Fig. 4). The CgA value before therapy correlated significantly with the TTD (Pearson correlation: = $\rho$ 0.67, *p* = 0.01). A higher CgA value resulted in a higher TTD. The linear dependence is illustrated in Fig. 5.

**Fig. 6:**
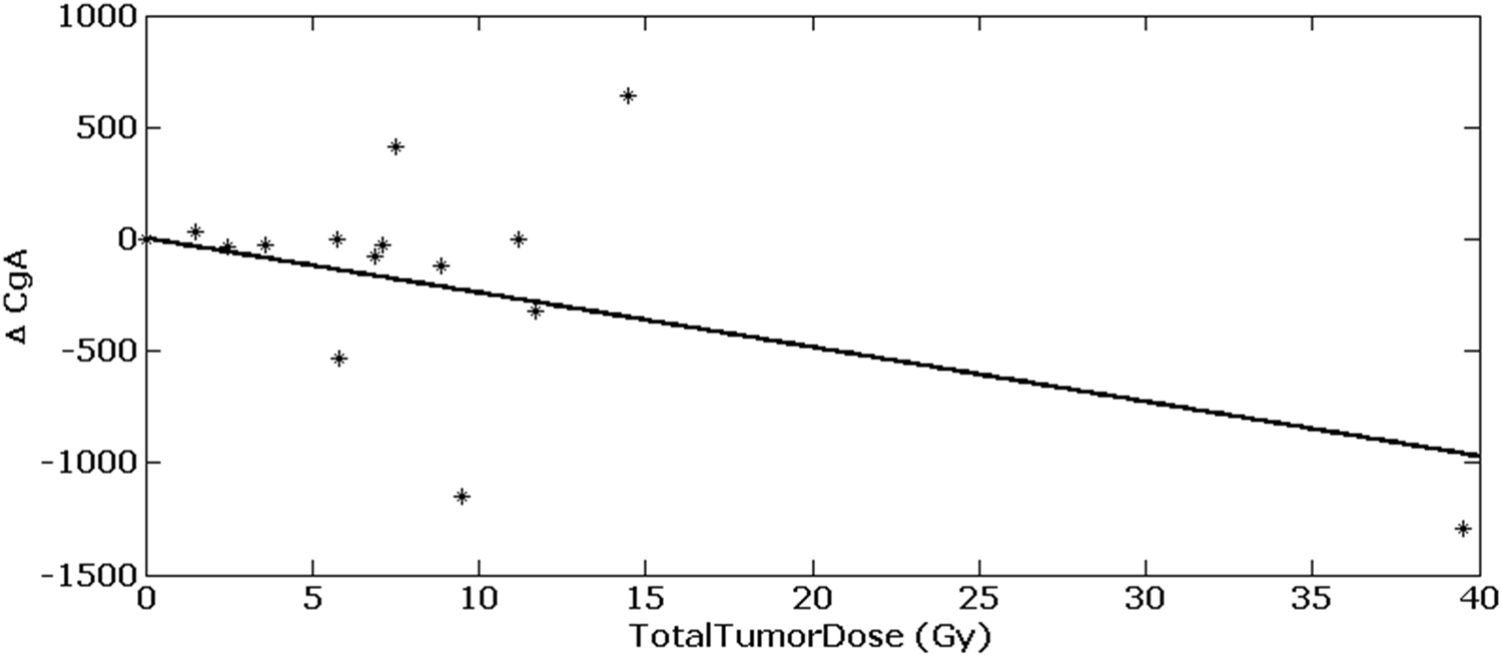
Difference in chromogranin A in dependency of TTD. (Pearson correlation: = $\rho$ − 0.54, *p* = 0.0451)

TTD values also significantly correlated with the difference between CgA values measured before and after therapy (Pearson-correlation: = $\rho$ − 0.54, *p* = 0.0451). A higher TTD is associated with a stronger decrease of CgA (see Fig. 6).

## Discussion

In this study, a three-dimensional dosimetry method was used to calculate a patient-specific voxel-wise dose map. The main advantages over the standard MIRD method are a voxel-wise, noise-free and patient-specific dose distribution, without assuming a standard phantom and scaling doses according to organ, e.g., kidney, masses. The voxel-wise method is very time consuming because of the full Monte Carlo simulation but yields appropriate results. The essential difference between the MIRD method and the proposed voxel-specific dose estimation is the following: The MIRD method performs a Monte Carlo simulation of radiation–matter interactions based on a mass density distribution as obtained from a standard human body phantom. In contrast, the proposed method receives information about patient-specific mass density distributions from related X-ray CT investigations as well as time-integrated radioactivity distributions from corresponding patient-specific SPECT investigations at various subsequent time points [18]. Thus, precise and patient-specific information about the spatial locations of relevant organs and tumor lesions enters the Monte Carlo simulations yielding precise radiation–matter interaction kernels. Note that this individualized information also encompasses knowledge about radiation from nearby contaminated organs. As previously reported, the difference in organ dose between the patient-specific Monte Carlo simulation and the standard human body phantom is 16.7% ± 12.8% [23]. In combination with the previously described method using the Mahalanobis distance and an alpha-stable test statistic, outlier-free average tumor doses can be obtained. The calculated dose value per injected activity of ^177^Lu-DOTATOC for the kidneys was on average 0.52 ± 0.20 mGy/MBq (0.20–1.62 mGy/MBq) and of 0.67 ± 0.41 mGy/MBq (0.20–1.62 mGy/MBq) for the spleen, and is in agreement with the value of 0.6 mGy/MBq and of 0.7 mGy/MBq, respectively, averaged over 59 patients and published in Ref. [14]. The mean effective half-live of ^177^Lu-DOTATOC in the kidneys of 67.4 h is close to the value of 63 h averaged over 30 patients reported in literature [24]. To date, the most extensively studied ^177^Lu-labeled somatostatin analogue is [^177^Lu-DOTA^0^, Tyr^3^]-octreotate (^177^Lu-DOTATATE) [9, 25]. Only a few studies directly compared ^177^Lu-DOTATATE and ^177^Lu-DOTATOC regarding tumor uptake and dosimetry in humans. Esser et al. [26] determined residence times for kidney, spleen and tumor in 7 patients for both agents demonstrating a favorable residence time ratio of tumor/kidney for ^177^Lu-DOTATATE. Kulkarni et al. [27] analyzed absorbed doses to tumor and kidneys in 22 patients who underwent an initial cycle of ^177^Lu-DOTATATE followed by a ^177^Lu-DOTATOC cycle. Their results suggested higher tumor–kidney ratios for ^177^Lu-DOTATOC. In the so far largest study by Schuchardt et al. [14], a total of 253 treatment naïve patients underwent ^177^Lu-PRRT with either ^177^Lu-DOTATATE (*n* = 185) ^177^Lu-DOTATOC (*n* = 59) or with ^177^Lu-DOTANOC (*n* = 9). Median absorbed doses to whole body, kidneys and spleen were significantly lower for ^177^Lu-DOTATOC compared to ^177^Lu-DOTATATE and ^177^Lu-DOTANOC; while, mean absorbed doses to tumor were comparable for ^177^Lu-DOTATOC and ^177^Lu-DOTATATE, whereas significantly lower for ^177^Lu-DOTANOC, resulting in the lowest dose to normal organs and the highest tumor–kidney ratio for ^177^Lu-DOTATOC.

However, there is, as yet, no evidence that the tumor absorbed dose could be predicted before administration of the ^177^Lu-labeled somatostatin analogue.

To the best of our knowledge, this is the first study that investigated the predictive value of tumor grade, tumor load and CgA values for the estimation of the tumor absorbed dose derived from Monte Carlo Simulations in patients with NETs who underwent ^177^Lu-DOTATOC therapy. We could demonstrate that the TTD is significantly higher in low- and intermediate-grade (G1–G2) tumors than compared to high-grade (G3) tumors. Most studies reported in the literature are conducted using ^177^Lu-DOTATATE PRRT and are limited to well-differentiated (G1–G2) tumors and only a small portion have included G3 tumors [28, 29] which are characterized by a short overall survival of 4–6 months [30] and are mainly treated with cytotoxic chemotherapy typically involving cisplatin/etoposide [31]. The reason for this is probably the higher expression of somatostatin receptors in well-differentiated tumors. Zamora et al. [32] investigated the immunohistochemical expression of somatostatin receptor-positive tumors and their metastases. They found that SSTRs are more frequently and homogeneously stained in well-differentiated than in poorly differentiated tumors. However, there is growing evidence that PRRT might also play a role in high-grade NETs. Nicolini et al. [33] reported the results of 33 patients with advanced gastroenteropancreatic neuroendocrine carcinomas who underwent 4–5 cycles of ^177^Lu-DOTATATE therapy. PRRT proved to be safe and effective, especially in patients with a Ki-67 index < 35%, with a disease control rate and progression-free survival comparable to standard therapy [34]. Their results are supported by the study of Thang et al. [29] in which 28 patients with grade three NETs underwent PRRT with or without radio-sensitizing chemotherapy. Patients with a Ki-67 index < 55% showed a median overall survival that was markedly longer than those reported from the NORDIC study in patients with G3 NETs who received first-line chemotherapy [30]. CgA is considered as the currently best available biomarker for the diagnosis of NETs since its serum plasma level is elevated in 90% of gut NETs [35]. Also, it has been proposed that CgA is more frequently elevated in well-differentiated tumors compared to poorly differentiated tumors of the midgut [36]. Furthermore, CgA is valuable in evaluating the efficacy of a broad range of therapies in NETs, including sandostatin therapy [37] or PRRT [38]. We could demonstrate that higher pretherapeutic CgA values significantly correlated with the TTD and higher TTD resulted in a significant decrease of CgA values. However, clinicians should be aware that an increase of CgA values following ^177^Lu-PRRT-therapy might be observed even in patients with an objective response or stable disease [39]. In our patient cohort, we could also observe patients who presented a high TTD and had increasing CgA values following therapy. These changes might occur due to radiation-induced inflammation or disease progression, and repeated measurements over time are necessary to differentiate between the two as suggested by Brabander et al. [39]. To validate our preliminary results regarding the role of histological and clinical parameters for the estimation of TTD derived from Monte Carlo Simulations, long-term follow-up and survival in treated patients should be evaluated in larger prospective multicenter trials.

Our study suffers from several limitations. First of all, results should be interpreted with caution due to the small number of patients. Also, the retrospective nature of this analysis has typical limitations, including possible biases stemming from patient referrals and treatments. Furthermore, this analysis was conducted as a single-center study. Long-term follow-up to determine response to therapy would have been preferable, but was not feasible. Due to the manual fashion of the ROI definition, exact position and size of the ROI are subject to intra- and inter-observer variability.

## Conclusion

These results suggest that Monte Carlo-based voxel-wise dosimetry is very promising for predicting the absorbed TTD based on histological and clinical parameters.
